# Cost‐effectiveness of lecanemab for early Alzheimer's disease in China

**DOI:** 10.1002/alz.71941

**Published:** 2026-10-27

**Authors:** Xingguang Zhao, Mingsong Xu, Hualin Chen, Zhixiang Lei, Qun Yu, Jun Liu, Wang Liao

**Affiliations:** ^1^ Department of Neurology Institute of Neuroscience The Second Affiliated Hospital, Key Laboratory of Neurogenetics and Channelopathies of Guangdong Province and the Ministry of Education of China Guangzhou Medical University Guangzhou China; ^2^ School of Public Health Guangzhou Medical University Guangzhou China; ^3^ The Second Affiliated Hospital Guangzhou Medical University Guangzhou China

**Keywords:** Alzheimer's disease, cost‐effectiveness analysis, dementia, lecanemab, Markov model, value‐based pricing

## Abstract

**BACKGROUND:**

Although lecanemab has demonstrated clinical efficacy in early Alzheimer's disease (AD), its cost‐effectiveness in China remains uncertain. This study evaluated the long‐term cost‐effectiveness of lecanemab in Chinese patients from a societal perspective and estimated its value‐based price (VBP).

**METHODS:**

A Markov state transition model based on Chinese real‐world data was developed to compare the long‐term health progression and economic outcomes of lecanemab combined with standard of care (SoC) versus SoC alone.

**RESULTS:**

Compared to SoC alone, the lecanemab combination therapy gained an additional 1.2432 Quality‐adjusted life years (QALYs) at an incremental cost of 463,228 Chinese yuan (CNY), resulting in an incremental cost‐effectiveness ratio (ICER) of 372,609 CNY/QALY (≈ 52,783 USD/QALY). To achieve cost‐effectiveness within the willingness‐to‐pay threshold, the price of a single vial of lecanemab must be reduced by at least 18.0%.

**DISCUSSION:**

Lecanemab can provide health benefits to Chinese patients with early AD, but it is not cost‐effective under the current pricing system.

## Highlights

- This is the first real‐world cost‐effectiveness analysis of lecanemab in China.
- We developed a societal‐perspective Markov model.
- Lecanemab improved quality‐adjusted life years but needs a price cut of at least 18% to be cost‐effective.
- Findings support value‐based pricing and reimbursement decisions.

## 1 INTRODUCTION

Alzheimer's disease (AD) is the leading cause of dementia worldwide.^1^ As the disease progresses, patients experience increasing care needs and health‐care resource use.^2^ With rapid population aging, AD has become a major public health challenge. Approximately 55 million people worldwide are living with dementia, of whom nearly 70% are affected by AD.^3^ China bears one of the largest dementia burdens globally, with > 13 million individuals living with AD and other dementias in 2019.^4^

Beyond its impact on health and quality of life, AD imposes a substantial economic burden on patients, families, and health‐care systems.^2^ The global cost of AD and other dementias is projected to reach US$1 trillion by 2030.^5^ China bears the largest national economic burden of dementia worldwide, with formal care costs estimated at US$205.94 billion in 2025.^6^

From a societal perspective, the economic burden of AD and other dementias extends beyond direct medical expenditures to include direct non‐medical costs and indirect costs.^7^ Informal care, predominantly provided by family members, is a major component of dementia care and contributes substantially to the overall socioeconomic burden.^5^ Moreover, cost composition varies by disease stage: direct medical costs are relatively higher in early stages, whereas social care and informal caregiving costs increase substantially in moderate and severe stages.^8^ In China, most individuals with dementia are cared for within the home by family members.^9^ Therefore, informal caregiving and related productivity losses account for a large share of the societal burden, highlighting the importance of a societal perspective in evaluating AD interventions.

Current therapeutic strategies for AD can be broadly categorized into symptomatic treatments and disease‐modifying therapies (DMTs). Conventional symptomatic medications, primarily including cholinesterase inhibitors and memantine, do not alter disease progression.^10^ In recent years, monoclonal antibodies targeting amyloid beta (Aβ) have emerged as a promising therapeutic approach for AD.^11^ Lecanemab selectively binds to amyloid protofibrils and promotes plaque clearance, thereby slowing disease progression.^12^ It was approved in the United States in 2023 and in China in 2024,^13^, ^14^ and demonstrated significant clinical benefits in the phase III CLARITY AD trial,^15^ with consistent findings reported in Chinese populations.^16^

**RESEARCH IN CONTEXT**

1. **Systematic review**: We reviewed the literature using PubMed and reference lists of relevant studies. Previous studies have evaluated the cost‐effectiveness of lecanemab in the United States, Japan, Sweden, and South Korea. However, evidence based on Chinese real‐world data and a societal perspective remains limited.
2. **Interpretation**: Using Chinese real‐world data, this study found that lecanemab provides additional health benefits for patients with early Alzheimer's disease but is unlikely to be cost‐effective in China at its current price. A substantial price reduction would be required to achieve cost‐effectiveness under the Chinese willingness‐to‐pay threshold.
3. **Future directions**: Future studies should incorporate larger real‐world cohorts, longer follow‐up periods, and China‐specific data on long‐term care costs, caregiver burden, and health utilities. Continued evaluation will be important as additional evidence on long‐term treatment effectiveness becomes available.

Although lecanemab has demonstrated clinical benefits, its affordability and long‐term cost‐effectiveness remain controversial. Treatment requires long‐term administration, imaging monitoring, and management of treatment‐related adverse events, while high acquisition costs may impose substantial pressure on health‐care systems and societal resource allocation. In recent years, several health economic evaluations across countries have assessed the cost‐effectiveness of lecanemab. However, most studies have suggested that, at current pricing levels, the incremental cost‐effectiveness ratio (ICER) exceeds commonly accepted willingness‐to‐pay (WTP) thresholds,^17^, ^18^, ^19^, ^20^ and that cost‐effectiveness may only be achieved under substantial price reductions.^17^

However, existing economic evaluations of lecanemab have largely relied on models based on Western populations, with limited consideration of China‐specific disease progression and long‐term care patterns. In addition, many studies have adopted a health‐care payer perspective and have not comprehensively incorporated societal costs related to long‐term care, informal caregiving, and institutional care. As AD progresses, patients transition from community to institutional care, with substantial differences in costs and utilities across settings. Real‐world factors such as treatment discontinuation and maintenance dosing may further influence long‐term cost‐effectiveness.

Therefore, using real‐world data from Chinese patients with early AD, we developed a Markov model to evaluate the long‐term costs and health outcomes associated with lecanemab treatment from a societal perspective. The model incorporated baseline characteristics and disease progression parameters derived from real‐world Chinese patients and considered both community‐based and institutional care settings. Key factors, including direct and indirect costs, treatment discontinuation, maintenance dosing adjustments, and adverse events, were incorporated to reflect routine clinical practice. The findings aim to inform health care decision making and resource allocation in AD management in China and provide evidence for future economic evaluations of DMTs in similar settings.

## 2 METHOD

### 2.1 Study population

This economic evaluation was based on real‐world data from patients with early AD who received lecanemab treatment at several tertiary hospitals in South China. The study population included patients with mild cognitive impairment (MCI) due to AD and those with mild AD dementia. All participants received lecanemab treatment and underwent baseline demographic and clinical assessments. AD‐related Aβ pathology was confirmed by amyloid positron emission tomography (Aβ PET) or cerebrospinal fluid (CSF) testing. Patients with contraindications to magnetic resonance imaging (MRI), such as metallic implants or claustrophobia, or those without evidence of Aβ positivity were excluded. In addition, patients with severe visual or hearing impairment that precluded cognitive assessment were also excluded.

A total of 108 patients with early AD were included in the study, comprising 52 patients with MCI (48.1%) and 56 patients with mild AD dementia (51.9%). The mean age of the cohort was 71.0 ± 9.1 years, and 57.4% were female. The mean body weight was 59.49 ± 16.73 kg. The mean baseline Clinical Dementia Rating Sum of Boxes (CDR‐SB) score was 4.39 ± 2.35, including 2.37 ± 1.16 in the MCI group and 6.27 ± 1.44 in the mild AD group. Apolipoprotein E ε4 (*APOE* ε4) carriers accounted for 53.7% of the study population. Detailed baseline demographic and clinical characteristics are presented in Table 1.

**TABLE 1:** Baseline characteristics of study population.

Characteristics	Overall (*N* = 108)	MCI (*N* = 52)	Mild AD (*N* = 56)
**Age (years), mean (SD)**	71.0 (9.1)	69.8 (10.3)	72.1 (7.6)
**Sex, *n* (%) ^a^**			
Female	62 (57.4%)	32 (61.5%)	30 (53.6%)
Male	46 (42.6%)	20 (38.5%)	26 (46.4%)
Weight (kg), mean (SD)	59.49 (16.73)	61.85 (22.00)	57.29 (9.57)
**Education, *n* (%) ^a^**			
Illiterate	2 (1.9%)	0 (0.0%)	2 (3.6%)
< 6 years	23 (21.3%)	12 (23.1%)	11 (19.6%)
6–12 years	50 (46.3%)	25 (48.1%)	25 (44.6%)
> 12 years	33 (30.6%)	15 (28.8%)	18 (32.1%)
***APOE* ε4 status, *n* (%)**			
Homozygote	10 (9.3%)	5 (9.6%)	5 (8.9%)
Heterozygote	48 (44.4%)	22 (42.3%)	26 (46.4%)
Non‐carrier	50 (46.3%)	25 (48.1%)	25 (44.6%)
**Baseline CDR‐SB, mean (SD)**	4.39 (2.35)	2.37 (1.16)	6.27 (1.44)

Abbreviations: AD, Alzheimer's disease; *APOE*, apolipoprotein E; CDR‐SB, Clinical Dementia Rating Sum of Boxes; MCI, mild cognitive impairment; SD, standard deviation.

The real‐world patient data collected in this study were primarily used to inform baseline model parameters, including age, disease stage, body weight, baseline cognitive status, and disease progression parameters associated with lecanemab treatment. In addition, longitudinal follow‐up data were used to estimate disease progression risks across different treatment stages, thereby better reflecting the natural disease course of patients with early AD in routine clinical practice in China.

### 2.2 Treatment strategies

This study compared two treatment strategies for patients with early AD: lecanemab plus standard of care (SoC) and SoC alone.

The SoC strategy included routine non‐pharmacological interventions and symptomatic pharmacological treatments for cognitive impairment, including cholinesterase inhibitors (e.g., donepezil) and the N‐methyl‐D‐aspartate (NMDA) receptor antagonist memantine. Treatment patterns were based on current Chinese and international clinical practice guidelines for AD.^21^

In the lecanemab group, patients received lecanemab in addition to SoC. According to the prescribing information and real‐world clinical practice, lecanemab was administered intravenously at a dose of 10 mg/kg every 2 weeks. During the initial treatment phase (first 18 months), all patients received the standard biweekly regimen. After 18 months, based on evidence from the open‐label extension (OLE) study and real‐world practice, 68% of patients were assumed to switch to a maintenance regimen administered every 4 weeks, while the remaining patients continued biweekly treatment.^22^, ^23^

The model further incorporated treatment discontinuation observed in real‐world clinical practice. All patients receiving lecanemab were assumed to face an all‐cause discontinuation risk, represented by a fixed discontinuation probability per cycle.^13^ In addition, lecanemab treatment was discontinued once patients progressed to moderate AD, after which they continued to receive SoC alone. For patients who discontinued treatment, a waning treatment effect was incorporated, whereby the risk of disease progression gradually returned toward that of SoC while retaining partial residual treatment benefits for a limited period.^24^

Furthermore, the model accounted for treatment‐related monitoring and adverse event management. Patients underwent MRI monitoring during the early treatment phase to assess the risk of amyloid‐related imaging abnormalities (ARIAs). The model also incorporated the impact of ARIAs on both costs and health‐related quality of life.

### 2.3 Model structure

A state‐transition Markov model was developed to simulate the long‐term clinical and economic outcomes of patients with early AD under different treatment strategies. To capture lifetime health outcomes, a time horizon of 20 years was adopted. A cycle length of 3 months was used to reflect the progressive nature of AD and the dosing schedule of DMT. In accordance with the Chinese Guidelines for Pharmacoeconomic Evaluations, all future costs and health outcomes were discounted at an annual rate of 5%.^25^ The analysis was conducted from a societal perspective, incorporating direct medical costs, direct non‐medical costs, and indirect costs.

The model structure was jointly defined by disease severity and care setting, resulting in nine mutually exclusive health states, including four disease stages (MCI, mild AD, moderate AD, and severe AD) across two care settings (community care and institutional care), together with death as an absorbing state^19^ (Figure 1). Disease severity was classified according to the CDR‐SB: MCI (0–4.5), mild AD (4.6–9.5), moderate AD (9.6–16.0), and severe AD (≥ 16.0).^17^

**FIGURE 1:**
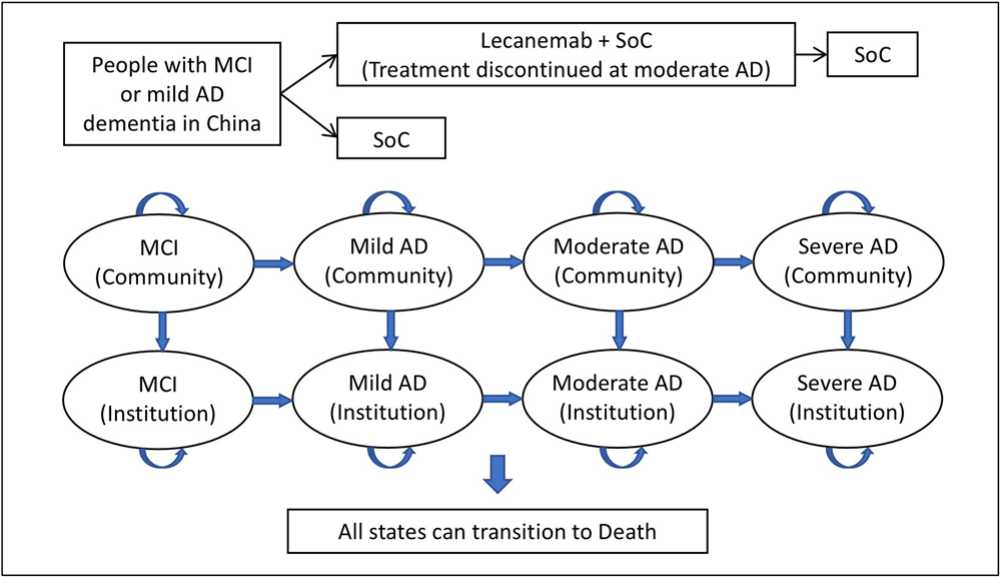
Integrated treatment pathway and Markov model structure. AD, Alzheimer's disease; MCI, mild cognitive impairment; SoC, standard of care

Patients were assumed to transition between health states during each cycle according to stage‐specific transition probabilities. Disease progression was modeled as movement toward more severe cognitive stages over time. Transitions from community care to institutional care were assumed to be unidirectional and irreversible.^18^ In addition, patients in any living health state could transition to death. Mortality risks were estimated by combining age‐specific all‐cause mortality rates from the Chinese population with disease stage–specific mortality hazard ratios associated with AD severity.

### 2.4 Model parameters

#### 2.4.1 Clinical and transition probabilities

Transition probabilities for patients receiving SoC were based on an Aβ+ natural history cohort to accurately serve as the reference for disease progression.^26^ For consistency with our unidirectional model structure, we classified these probabilities as progression or non‐progression, rather than allowing transitions to less severe stages or skipping multiple disease stages. Annual progression probabilities were converted into 3‐month transition probabilities and corresponding instantaneous transition rates required for the Markov model. To capture the long‐term benefits of DMTs, a two‐phase dynamic transition framework was developed for the lecanemab strategy by integrating real‐world evidence (RWE) with long‐term clinical trial data (Supplemental S1 in supporting information).

During the active treatment phase, disease progression parameters were estimated using longitudinal follow‐up data from the study cohort. First, a linear mixed model (LMM) incorporating random intercepts and random slopes was applied to estimate the annual rate of change in CDR‐SB scores among patients receiving lecanemab, effectively accounting for between‐patient differences in baseline disease severity and progression rates.^27^ The estimated slope was used to quantify the rate of disease progression. Subsequently, clinically established CDR‐SB thresholds defining transitions between disease stages were applied to estimate the time‐to‐progression (TTP) from the current stage to the next disease stage. Assuming an exponential distribution, TTP was converted into hazard rates and subsequently transformed into 3‐month transition probabilities for model implementation. Comparison to the corresponding Aβ + SoC progression rate yielded a relative treatment effect, which was then applied to the SoC transition probabilities to derive the treatment‐adjusted progression rates.

Once patients progressed to moderate AD, a waning treatment effect was incorporated into the model. Based on long‐term follow‐up reports from the phase 2 Study 201 open‐label extension,^28^ the disease‐delaying effect of lecanemab was assumed to persist after treatment discontinuation and gradually decline over time.^24^ Instead of ceasing immediately, the treatment effect was assumed to decline linearly, reaching 39% of the active‐treatment effect after 1 year and fully reverting to the natural disease progression level observed under the SoC group by the end of the second year.

Baseline mortality was derived from age‐specific all‐cause mortality rates reported in the China Population Census Yearbook and converted into age‐dependent cycle‐specific mortality probabilities^29^, ^30^ (Table S1 in supporting information). Given the elevated mortality risk among patients with AD, mortality probabilities were further adjusted using disease stage–specific hazard ratios (HRs). Treatment‐related adverse events associated with lecanemab, including ARIAs and infusion‐related reactions, were informed by real‐world clinical data from South China to better reflect treatment patterns among Chinese patients.^15^

To more accurately capture differences in resource use and health outcomes across care settings, each disease stage (MCI, mild AD, moderate AD, and severe AD) was further stratified into community care and institutional care states.^31^ Transition probabilities from community care to long‐term institutional care were obtained from published literature (Table 2).

**TABLE 2:** Key model input parameters for the base‐case model.

Parameter	Base‐case value	Range	Distribution	Sources
**General settings**				
Time horizon (years)	20	–		Assumption
Cycle length (months)	3	–		18
Annual discount rate (%)	5	4–6		25
**Cohort baseline characteristics**				
Starting age (years)	71	61.9–80.1	Normal	Cohort
MCI, *N* (%)	52 (48.1)	–		Cohort
Mild AD, *N* (%)	56 (51.9)	–		Cohort
Baseline institutionalization rate (%)	2	1.6–2.4	Beta	31
Mean patient weight (kg)	59.49	42.76–76.22	Normal	Cohort
**Per‐cycle disease progression probabilities (SoC)**				
MCI to mild AD	0.0628	0.0502–0.0754	Beta	26
Mild AD to moderate AD	0.1182	0.0946–0.1418	Beta	26
Moderate AD to severe AD	0.1256	0.1005–0.1507	Beta	26
**Active treatment absolute probabilities**				
MCI to mild AD	0.0162	0.0116–0.0206	Beta	Cohort
Mild AD to moderate AD	0.0311	0.0224–0.0395	Beta	Cohort
All‐cause discontinuation rate per cycle (%)	3.41	2.73–4.09	Beta	13
**Absolute probabilities of waning effect**				
MCI to mild AD	0.0423	0.0404–0.0442	Beta	24, 28
Mild AD to moderate AD	0.0805	0.0768–0.0840	Beta	24, 28
Moderate AD to severe AD	0.0856	0.0817–0.0894	Beta	24, 28
**Mortality risk (HR vs. general population)**				
MCI	1.61	1.49–1.74	Lognormal	29
Mild AD	2.23	1.77–2.82	Lognormal	29
Moderate AD	3.1	2.47–3.89	Lognormal	29
Severe AD	4.98	3.85–6.44	Lognormal	29
**Adverse events of lecanemab (first 6 months)**				
Overall probability (%)	9.20	7.36–11.04	Beta	16
Probability of mild ARIA (%)	6.90	5.52–8.28	Beta	16
Probability of moderate ARIA (%)	1.90	1.52–2.28	Beta	16
Probability of severe ARIA (%)	0.40	0.32–0.48	Beta	16
**Institutionalization probabilities (per cycle)**				
MCI to institution	0.0050	0.0040–0.0060	Beta	45
Mild AD to institution	0.0095	0.0076–0.0114	Beta	17
Moderate AD to institution	0.0289	0.0231–0.0347	Beta	17
Severe AD to institution	0.0721	0.0577–0.0865	Beta	17
**Health state utilities**				
MCI (community)	0.988	0.790–0.999	Beta	33, 34
MCI (institution)	0.829	0.663–0.995	Beta	33, 34
Mild AD (community)	0.922	0.738–0.999	Beta	33, 34
Mild AD (institution)	0.763	0.610–0.916	Beta	33, 34
Moderate AD (community)	0.821	0.657–0.985	Beta	33
Moderate AD (institution)	0.662	0.530–0.794	Beta	33
Severe AD (community)	0.595	0.476–0.714	Beta	34
Severe AD (institution)	0.436	0.349–0.523	Beta	34
Disutility: severe ARIA	0.14	0.112–0.168	Beta	36
**Caregiver disutility**				
MCI	0	–		20
Mild AD	0.036	0.029–0.043	Beta	20
Moderate AD	0.07	0.056–0.084	Beta	20
Severe AD	0.086	0.069–0.103	Beta	20
**Costs: SoC**				
MCI (community)	17,161	13,729–20,593	Gamma	37
MCI (institution)	12,759	10,207–15,311	Gamma	20, 37, 38
Mild AD (community)	50,474	40,379–60,569	Gamma	37
Mild AD (institution)	37527	30,022–45,032	Gamma	37, 38
Moderate AD (community)	64,722	51,778–77,666	Gamma	37
Moderate AD (institution)	48,120	38,496–57,744	Gamma	37, 38
Severe AD (community)	102,985	82,388–123,582	Gamma	37
Severe AD (institution)	76,569	61,255–91,883	Gamma	37, 38
**Costs: Lecanemab**				
Lecanemab unit price (per 200 mg vial)	2508	2006–3010	Gamma	Current price
Administration cost (MRI monitoring)	600	480–720	Gamma	Assumption

*Note*: All costs are converted and presented in 2026 Chinese Yuan (RMB).

Abbreviations: AD, Alzheimer's disease; ARIA, amyloid‐related imaging abnormality; HR, hazard ratio; MCI, mild cognitive impairment; MRI, magnetic resonance imaging; SoC, standard of care.

#### 2.4.2 Health state utilities

Quality‐adjusted life years (QALYs) were used as the primary measure of health outcomes in this study. Health‐related quality of life (HR‐QoL) was quantified using utility values derived from the EuroQol 5‐Dimension (EQ‐5D) instrument.^32^ Due to the lack of China‐specific utility estimates, preference was given to utility values reported in previous studies conducted in Asian populations, which used proxy‐based assessments to account for patients’ cognitive limitations.^33^, ^34^ Utility values were assigned according to both AD disease stage and care setting (community care versus institutional care).

In addition to patient utilities, caregiver disutility was incorporated from a societal perspective.^21^ Given the substantial psychological, physical, and social burden associated with long‐term caregiving for patients with AD, disease stage–specific caregiver disutility values were assigned and deducted from patient QALYs to estimate net health benefits. Furthermore, short‐term reductions in HR‐QoL associated with lecanemab‐related adverse events were incorporated into the model. Temporary utility decrements were applied to patients experiencing infusion‐related reactions or severe ARIAs and were modeled as one‐time disutilities within the corresponding cycle.^18^ Consistent with previous studies, all patients experiencing severe ARIAs were assigned a utility decrement of 0.14 for a duration of 12 weeks.^35^

#### 2.4.3 Cost inputs

The analysis was conducted from a societal perspective. Cost components included total societal costs associated with each disease stage, lecanemab treatment and monitoring costs, and adverse event management costs.

The total societal cost associated with each AD stage comprised direct medical costs, direct non‐medical costs, and indirect costs. Direct medical costs included expenditures incurred within the formal health‐care system, such as hospital care, medications, diagnostic procedures, and outpatient visits. Direct non‐medical costs included services provided outside the health‐care system, such as nursing home or long‐term care services, as well as transportation and food‐related expenses. Indirect costs included informal caregiving provided by family members or friends and productivity losses attributable to the disease.^36^

Annual costs for community‐dwelling patients with mild, moderate, and severe AD under SoC were obtained from a Chinese study on the economic burden of AD.^37^ This study adopted a health economic perspective and incorporated direct medical expenditures, including hospitalization, outpatient visits, and medication costs. Informal caregiving time and productivity losses were monetized using the opportunity cost approach to estimate the overall societal costs of AD.

As China‐specific estimates for societal costs in institutional care settings and the MCI stage are currently unavailable, additional assumptions were derived from published studies conducted in China and Japan. Previous studies reported that the total cost of community care was ≈ 1.345 times that of institutional care, while the societal cost of MCI was ≈ 34% of that of mild AD.^20^, ^38^ These proportional relationships were used to estimate annual costs for all health states included in the model.

All historical cost inputs were updated to 2025 Chinese yuan (RMB) values. Medical costs were adjusted via the national Healthcare Consumer Price Index (CPI), while informal care costs were adjusted via the national Wage Growth Index to align with the opportunity cost approach.^39^ These annualized costs were then adjusted to the 3‐month cycle length of the Markov model. For international comparability, a consistent exchange rate of USD 1 = CNY 7.0593 (December 2025 period average) was applied, as reported by the China Foreign Exchange Trade System.^40^

In addition to SoC‐related costs, the lecanemab strategy incorporated drug acquisition costs, intravenous infusion costs, and MRI monitoring costs. Drug dosing was calculated according to the recommended regimen of 10 mg/kg and the average body weight (59.49 kg) observed in the Chinese real‐world early AD cohort. During the initial treatment phase (first 1.5 years), patients were assumed to receive intravenous infusions every 2 weeks. During the maintenance phase, based on real‐world long‐term treatment patterns, 32% of patients were assumed to continue biweekly administration, whereas 68% switched to a reduced‐frequency regimen administered every 4 weeks, thereby reducing long‐term treatment expenditures.^23^

According to current safety monitoring recommendations for lecanemab, patients underwent regular MRI examinations during the early treatment phase to detect ARIAs. The model assumed two MRI examinations during the first cycle and one MRI examination during each of the second and third cycles, with the corresponding imaging costs included. When patients progressed to moderate AD, all lecanemab‐related treatment and monitoring costs were discontinued.

Based on current clinical practice and treatment recommendations in China, most patients experiencing mild‐to‐moderate ARIAs do not require specific pharmacological intervention. Instead, patients typically undergo MRI monitoring every 4 weeks until the event resolves or stabilizes. Given that the average duration of ARIA is ≈ 12 weeks, management of each ARIA event was assumed to require three additional MRI examinations, and the corresponding monitoring costs were incorporated into the model.^41^

### 2.5 Sensitivity analyses

To assess the robustness of the base‐case results and identify key drivers of the ICER, one‐way sensitivity analyses (OWSA) were conducted. All major model parameters, including baseline patient characteristics, disease progression transition probabilities, health utility values across disease stages and care settings, direct medical costs, and discount rates, were varied individually over their respective ranges while holding all other parameters constant.^42^

To evaluate the overall impact of parameter uncertainty on model outcomes, probabilistic sensitivity analyses (PSA) were performed using 1000 Monte Carlo simulation iterations. During each iteration, model parameters were randomly sampled from predefined probability distributions. Clinical transition probabilities, institutionalization rates, and health utility and disutility were assigned beta distributions, cost parameters were assigned gamma distributions, baseline patient characteristics were assigned normal distributions, and mortality risk hazard ratios were assigned lognormal distributions.^43^

For parameters supported by real‐world data, the corresponding 95% confidence intervals were used as uncertainty ranges. For cost, utility, and other transition parameters without explicitly reported variance, a ± 20% variation around the base‐case value was applied according to commonly adopted practices in pharmacoeconomic evaluations.

### 2.6 Scenario analyses

A series of scenario analyses were conducted to evaluate the robustness of the cost‐effectiveness results under alternative assumptions. These scenarios included: (1) alternative treatment durations, assuming lecanemab treatment for 18 months (corresponding to the clinical trial duration), 3 years, 4 years, or lifetime treatment; (2) alternative baseline disease states, assuming all patients entered the model at either the MCI stage or the mild AD stage; (3) alternative treatment‐effect and disease progression assumptions, including the use of the overall HR (0.69) reported in the CLARITY AD trial,^12^ replacement of SoC disease progression rates with estimates from alternative published studies, and construction of a bidirectional disease progression model; (4) alternative structural assumptions on long‐term and maintenance efficacy, including a scenario with absolutely no residual disease‐delaying benefit upon treatment discontinuation, and a scenario assuming halved treatment efficacy during the reduced‐frequency maintenance phase; (5) an internal validation scenario substituting the base‐case parameters with observed survival analysis–derived progression probabilities; (6) alternative health utility sets derived from studies conducted in South Korea and Sweden;^18^, ^19^ (7) an increased institutional care use scenario, in which the initial proportion of patients receiving institutional care and stage‐specific institutionalization probabilities were updated using alternative estimates;^44^, ^45^ (8) an insurance reimbursement scenario based on a commercial supplementary insurance program in Guangzhou, under which 50% of outpatient drug costs and 70% of inpatient drug costs were reimbursed after an annual deductible of RMB 16,000.

All scenario analyses adopted the same model structure and parameter framework as the base‐case analysis. The ICER was used as the primary outcome measure. Results were compared to the WTP threshold of three times China's per‐capita gross domestic product (GDP) in 2025 (RMB 298,995 per QALY gained). Detailed information is presented in Supplemental S2 in supporting information.

### 2.7 Threshold analysis and value‐based pricing

Threshold analyses were performed to estimate the value‐based price (VBP) of lecanemab within the Chinese health‐care system. Using the incremental net monetary benefit (INMB) framework, the drug price corresponding to an INMB of zero was identified.^20^, ^28^, ^46^ This threshold represents the maximum justifiable unit price of lecanemab at a WTP threshold of three times China's 2025 per‐capita GDP (RMB 298,995 per QALY gained), with an additional evaluation under the stricter threshold of one time the per‐capita GDP (RMB 99,665/QALY).^20^ Based on the recommended dosing regimen for a patient with an average body weight of 59.49 kg, we further estimated the corresponding value‐based annual treatment cost during the first year of therapy. In addition, the minimum price reduction required for lecanemab to become cost‐effective under the Chinese WTP threshold was calculated.

## 3 RESULTS

### 3.1 Base‐case analysis

Over the 20‐year lifetime simulation horizon, the lecanemab + SoC strategy generated greater health benefits than SoC alone. Patients receiving lecanemab remained in the higher functioning disease stages for a longer period, spending an additional 17 months in the MCI stage and 16.3 months in the mild AD stage. Correspondingly, time spent in more advanced disease stages was reduced, with expected durations in the moderate AD and severe AD stages decreasing by 1.4 months and 18.2 months, respectively (Figure 2).

**FIGURE 2:**
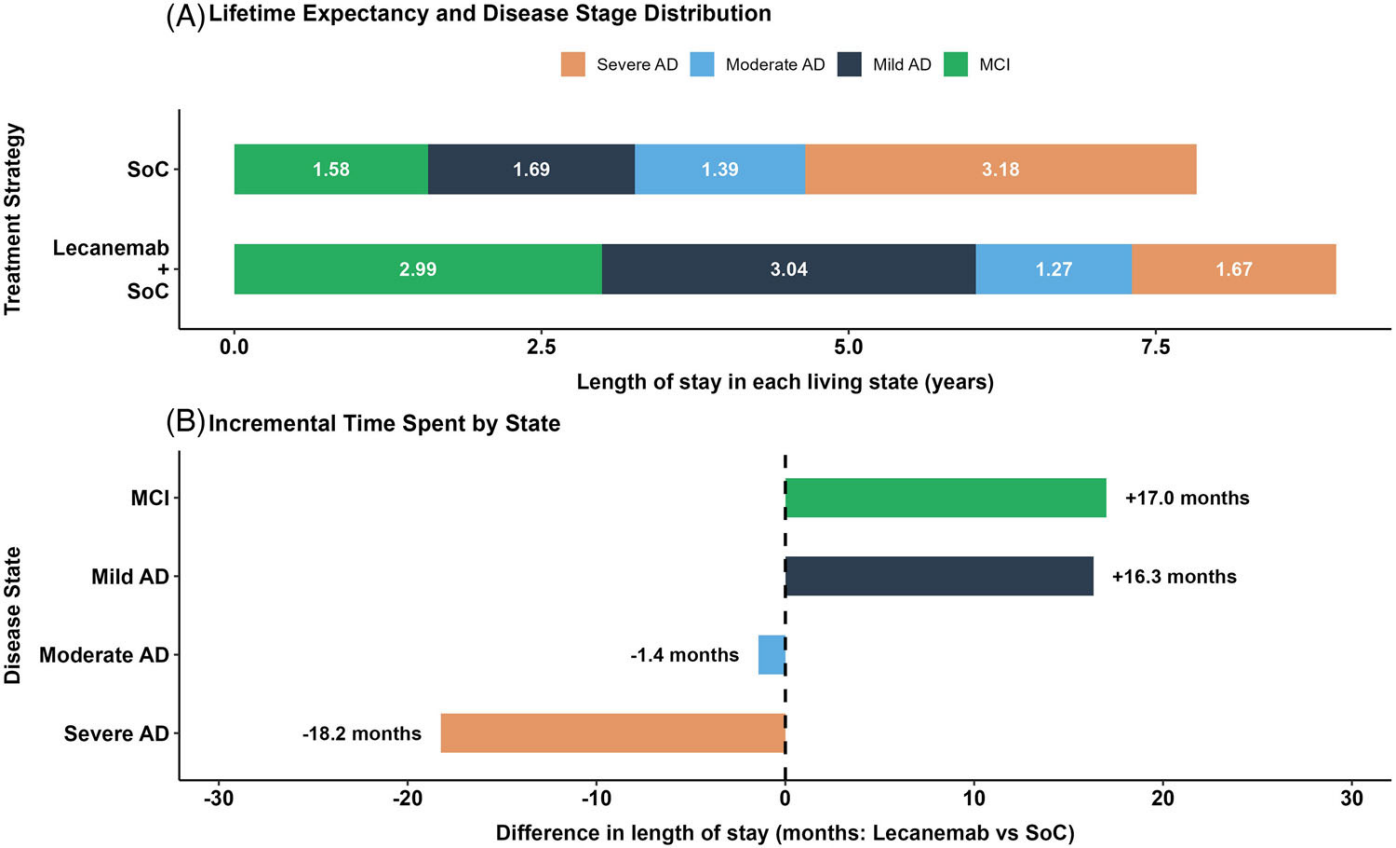
Distribution of life‐years across disease stages and incremental differences in length of stay. A, Estimated lifetime expectancy partitioned by clinical stage for standard of care and lecanemab. B, Incremental time spent by state. AD, Alzheimer's disease; MCI, mild cognitive impairment; SoC, standard of care

These health gains were accompanied by a substantial increase in total health‐care expenditure. The lifetime cost per patient in the SoC group was RMB 360,776, with a total of 4.3666 QALYs gained. In contrast, the lecanemab + SoC strategy increased lifetime costs to RMB 824,004 and generated 5.6098 QALYs. Compared to SoC alone, lecanemab treatment resulted in an additional RMB 463,228 in costs and 1.2432 incremental QALYs, yielding an ICER of RMB 372,609 (USD 52,783) per QALY gained (Table 3). Using the commonly adopted Chinese WTP threshold of three times the national GDP per capita (RMB 298,995/QALY), the estimated ICER substantially exceeded the threshold. Therefore, at its current price and from a societal perspective, lecanemab is unlikely to be considered cost‐effective for patients with early AD in China.

**TABLE 3:** Base‐case cost‐effectiveness analysis results.

Strategy	Total costs (CNY)	Total QALYs	Incremental costs (CNY)	Incremental QALYs	ICER (CNY/QALY)
SoC	360,776	4.3666			
Lecanemab + SoC	824,004	5.6098	463,228	1.2432	372,609

Abbreviations: CNY, Chinese Yuan; ICER, incremental cost‐effectiveness ratio; QALY, quality‐adjusted life year; SoC, standard of care.

### 3.2 Sensitivity analyses

The OWSA demonstrated that the ICER of lecanemab + SoC remained above the Chinese WTP threshold across the vast majority of plausible parameter ranges. A tornado diagram (Figure 3) identified the most influential parameters: patient mean weight, starting age, lecanemab unit price, and the natural disease progression rates under SoC.

**FIGURE 3:**
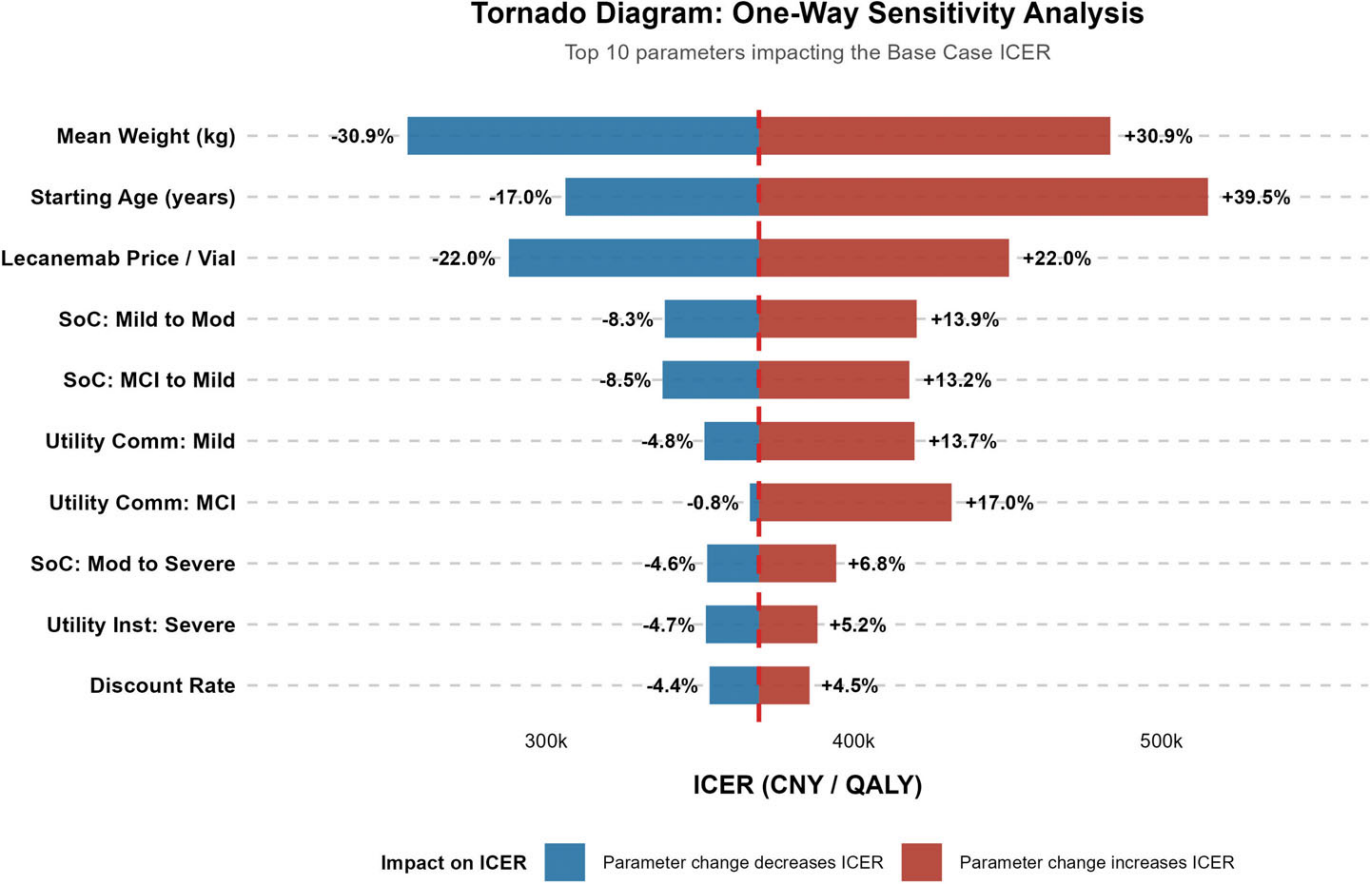
Tornado diagram of one‐way sensitivity analysis. CNY, Chinese yuan; ICER, incremental cost‐effectiveness ratio; MCI, mild cognitive impairment; QALY, quality‐adjusted life year; SoC, standard of care

Specifically, reducing the mean weight to its lower bound (42.76 kg) or the lecanemab unit price by 20% (RMB 2006/vial) yielded ICERs of RMB 254,925/QALY and RMB 287,848/QALY, respectively, both falling below the WTP threshold. The high sensitivity to weight is driven by the assumption of exact dose splitting in the OWSA, by which lower body weight proportionally reduces drug acquisition costs. Variations in all other parameters resulted in ICERs ranging from ≈ RMB 337,804 to RMB 515,094/QALY, consistently exceeding the threshold (Table S2 in supporting information).

PSA confirmed that, compared to SoC alone, lecanemab treatment was associated with a mean incremental cost of RMB 450,135 (95% confidence interval [CI]: RMB 285,222–657,332) and a mean incremental gain of 1.2289 QALYs (95% CI: 0.7137–1.7508). The median ICER across 1000 Monte Carlo simulations was RMB 366,433/QALY. Although lecanemab consistently generated additional health benefits, these gains were accompanied by substantially higher costs. The cost‐effectiveness acceptability curve (CEAC; Figure 4) illustrates the probability that lecanemab is cost‐effective across a range of WTP thresholds. At the current WTP threshold of RMB 298,995/QALY, the probability of lecanemab being cost‐effective remains highly limited, with the probability curve only approaching 50% as the threshold increases to meet the median ICER of RMB 366,433/QALY (Table S3 in supporting information).

**FIGURE 4:**
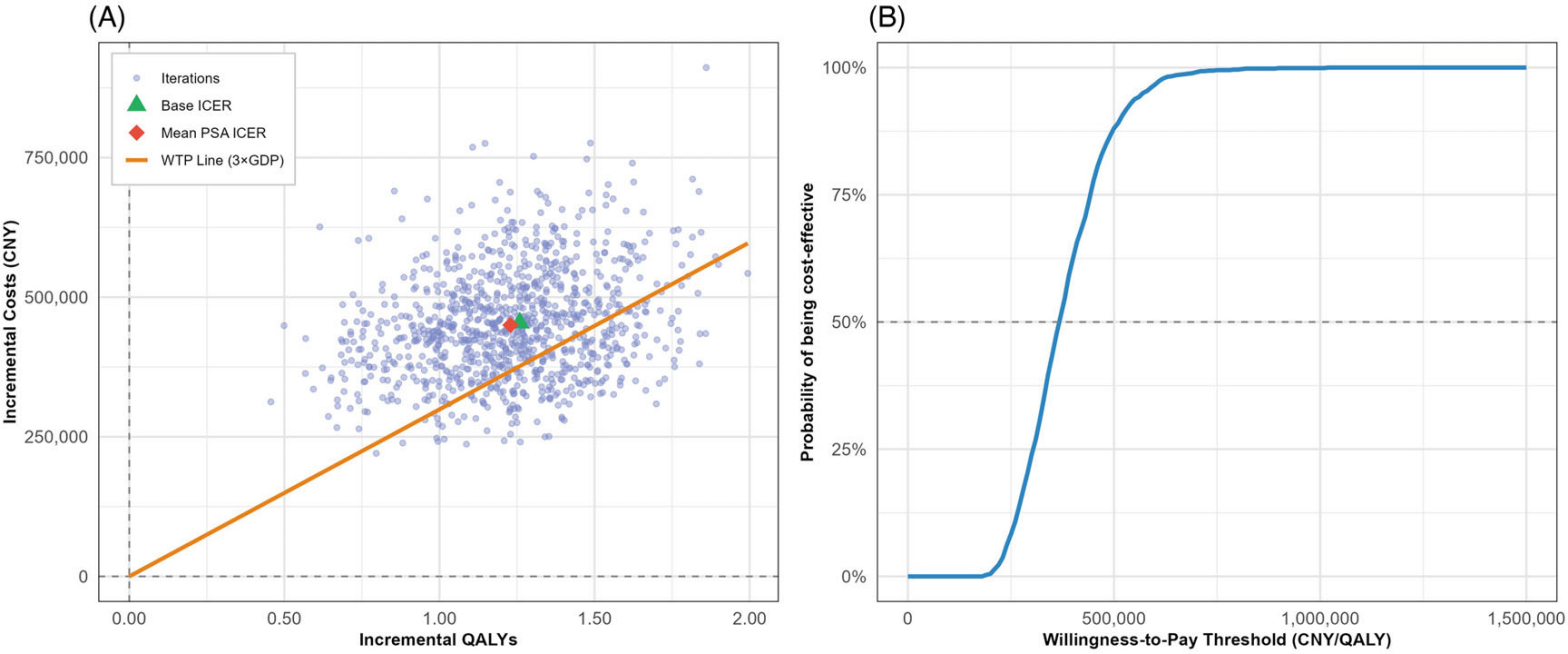
A, Cost‐effectiveness plane. B, Cost‐effectiveness acceptability curve. CNY, Chinese yuan; GDP, gross domestic product; ICER, incremental cost‐effectiveness ratio; PSA, probabilistic sensitivity analyses; QALY, quality‐adjusted life year; SoC, standard of care; WTP, willingness to pay

### 3.3 Scenario analyses

The results of the scenario analyses were generally consistent with those of the base‐case analysis, with lecanemab + SoC exceeding the Chinese WTP threshold in most scenarios (Table S4 in supporting information).

Under alternative treatment duration scenarios, longer treatment durations were associated with greater QALY gains but also substantially higher costs. The ICER increased from RMB 228,288/QALY for the 18‐month treatment scenario to RMB 419,130/QALY under the lifetime treatment scenario.

Analyses stratified by baseline disease stage showed that the ICER was lower when all patients entered the model at the MCI stage (RMB 370,555/QALY). In contrast, when all patients were assumed to initiate treatment at the mild AD stage, the ICER increased to RMB 476,509/QALY, suggesting that earlier treatment initiation was associated with greater QALY gains (due to more time preserved in the highest utility state) and more favorable cost‐effectiveness.

In the treatment‐effect and disease progression scenarios, applying the more conservative efficacy estimate from the CLARITY AD trial (HR = 0.69) increased the ICER to RMB 743,142/QALY, further reducing cost‐effectiveness. When alternative SoC disease progression rates from published literature were applied, the ICER increased markedly to RMB 1,122,389/QALY, as the slower baseline disease progression left a smaller window for the relative treatment effect. When both alternative disease progression rates and bidirectional disease transitions were incorporated into the model, the ICER increased to RMB 877,522/QALY.

Under the new conservative structural assumptions penalizing the drug's long‐term benefits assuming no residual benefit upon discontinuation or halved efficacy during maintenance, the ICERs increased to RMB 480,753/QALY and RMB 440,273/QALY, respectively. Furthermore, substituting the base‐case parameters with raw, survival analysis–derived transition probabilities yielded an ICER of RMB 573,940/QALY.

Using alternative health utility inputs from South Korean and Swedish studies resulted in ICERs of RMB 479,543/QALY and RMB 418,019/QALY, respectively, indicating that the results were relatively insensitive to variations in health utility estimates. And in the increased institutional care use scenario, the ICER increased to RMB 375,771/QALY.

In the insurance reimbursement scenario, incorporating supplementary commercial insurance coverage significantly reduced patients’ financial burden, and lecanemab + SoC became cost‐effective, with the ICER decreasing to RMB 187,677/QALY, falling well below the Chinese WTP threshold.

Overall, although the magnitude of the ICER varied across different assumptions, most scenario analyses showed that lecanemab + SoC remained above the study‐specific WTP threshold of RMB 298,995/QALY (three times China's per‐capita GDP), indicating limited cost‐effectiveness under a wide range of clinical and economic conditions, unless treatment duration is strictly capped or substantial supplementary insurance coverage is available.

### 3.4 Value‐based pricing

Threshold analysis indicated that, at a WTP threshold of RMB 298,995/QALY, the current market price of lecanemab (RMB 2508 per vial) was not cost‐effective. As shown in Figure 5, the INMB of lecanemab versus SoC increased linearly as the unit price decreased. When the price per vial decreased to RMB 2057, the INMB curve intersected the zero line, representing the VBP of lecanemab. Based on this VBP, the reasonable annual treatment cost for a standard nearly 60 kg patient would be limited to RMB 160,467 in the first year. Furthermore, because the China Guidelines for Pharmacoeconomic Evaluations also suggest a stricter WTP threshold of 1× per‐capita GDP (RMB 99,665/QALY), under this strict standard, the VBP would drop significantly to RMB 836.75 per vial, corresponding to a first‐year annual treatment cost of RMB 65,267. Overall, to achieve cost‐effectiveness for market access in China, the price of lecanemab would need to be reduced by at least 18.0% from its current level to meet the 3× GDP upper bound, and by up to 66.6% to meet the strict 1× GDP standard.

**FIGURE 5:**
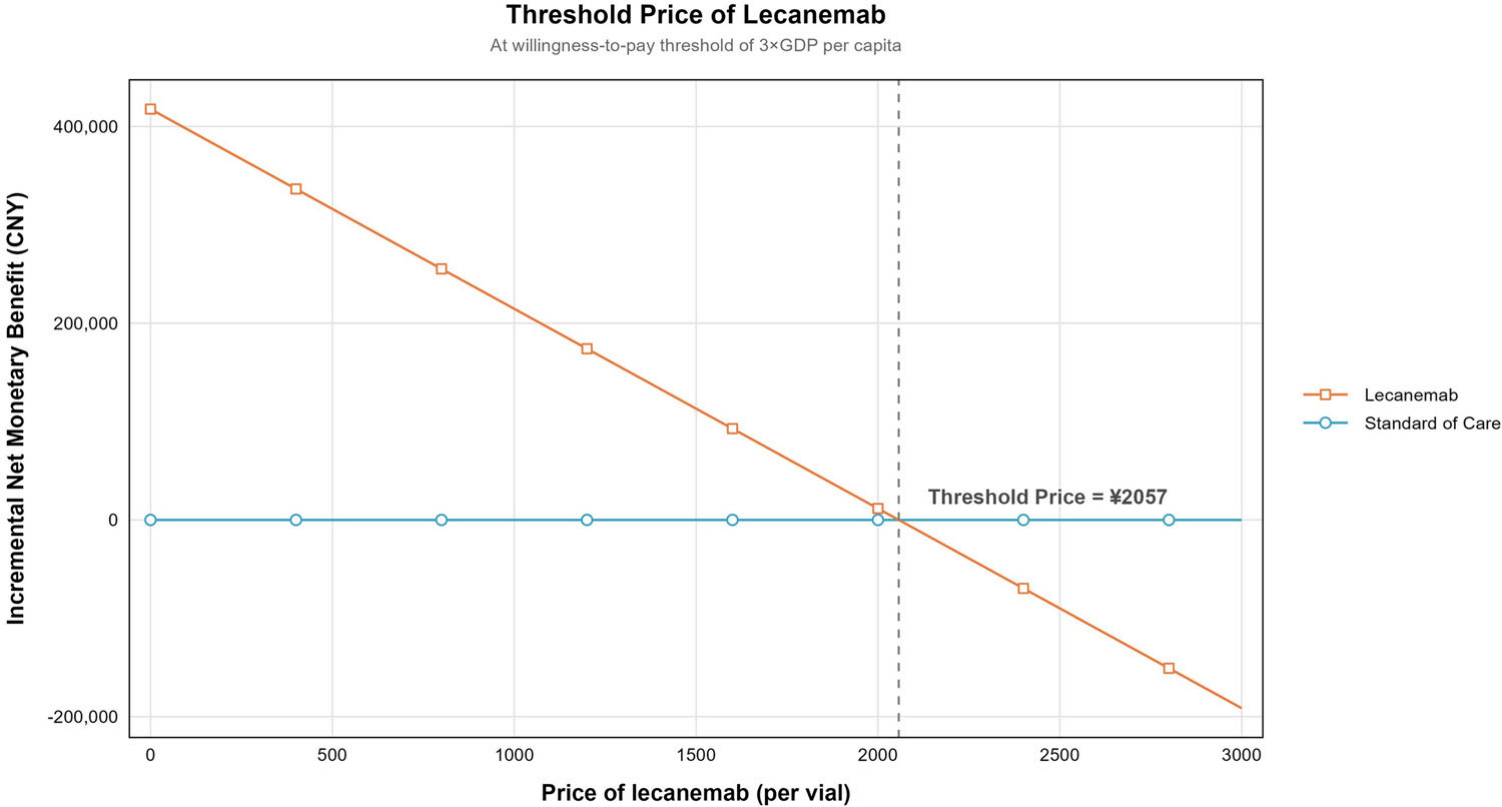
Threshold analysis of the value‐based price for lecanemab. CNY, Chinese yuan; GDP, gross domestic product

## 4 DISCUSSION

This study is the first to conduct a long‐term cost‐effectiveness analysis of lecanemab treatment for patients with early AD in China from a societal perspective based on Chinese real‐world data. Unlike most previous studies that relied on clinical trial data from the United States, this study incorporated key factors such as real‐world information on elderly AD patients in China and longitudinal disease progression data, which are representative of the potential eligible population and highly consistent with the baseline characteristics of a recent multicenter study,^47^ aiming to more realistically simulate the long‐term health and economic value of DMT in the context of Chinese clinical practice, and providing a reference for economic evaluations of DMTs for AD in low‐ and middle‐income countries.

The results show that, over a lifetime horizon, lecanemab + SoC provides modest health gains compared to SoC alone; however, these benefits are accompanied by substantially increased long‐term treatment costs, resulting in an ICER of RMB 372,609/QALY, which is higher than the commonly used WTP threshold in China, defined as three times per capita GDP (RMB 298,995/QALY). Further OWSA, PSA, and multiple scenario analyses consistently indicated that, at the current price level, lecanemab does not demonstrate clear economic value in China.

These findings are broadly consistent with existing pharmacoeconomic evaluations of anti‐Aβ monoclonal antibodies internationally. Many countries have similarly shown that, although lecanemab can delay disease progression and improve outcomes for patients and caregivers, it remains not cost‐effective at current pricing levels.^48^, ^49^, ^50^ For example, a study from South Korea reported an increase of 0.46 QALYs, with an ICER of ≈ USD 137,262/QALY. Other earlier evaluations from the United States and Sweden also reported relatively conservative incremental QALY gains and concluded that the therapy's high costs far exceeded local WTP thresholds.^18^, ^19^, ^41^ In contrast, our finding of a more substantial health benefit aligns closely with recent evidence from Japan, which highlighted that lecanemab significantly extended the time spent in early AD, thereby maintaining patient quality of life and reducing medical and long‐term care resource usage.^51^ That study reported an incremental gain of 1.31 QALY for MCI patients, closely mirroring the clinical benefits observed in our model. Yet, despite these QALY gains, the therapy still exceeded acceptable cost‐effectiveness thresholds. Collectively, these findings suggest that the economic challenges associated with anti‐amyloid therapies are not unique to China but represent a global issue.

Baseline disease progression and treatment effect parameters significantly influence the economic evaluation of lecanemab. We retained the Chinese cohort in the base case for critical policy relevance, as the CLARITY AD trial lacked standalone efficacy data for the Chinese mainland. Our cohort provides real‐world evidence of disease progression in routine Chinese clinical practice. This is supported by recent data demonstrating remarkable cognitive stabilization (an adjusted mean CDR‐SB increase of 0.30 at 12 months), reflecting noticeably slower progression than the global CLARITY AD trial.^47^ We acknowledge that measuring this real‐world trajectory against natural history baseline yields a larger disease‐slowing effect. Consequently, this base‐case choice explicitly biases the evaluation in favor of lecanemab, generating larger incremental QALY gains and a more optimistic ICER. Importantly, the VBP estimates proposed in this study rest inherently on this optimistic assumption. Applying the more conservative overall trial HR of 0.69 in scenario 4 increased the ICER to CNY 743,142/QALY. This variation implies lecanemab may exhibit stronger real‐world efficacy in Chinese patients, yet confirms the therapy remains not cost‐effective under either assumption.

Furthermore, the choice of SoC progression significantly impacts model outcomes. Substituting the biomarker‐confirmed SoC baseline with progression rates from a general, non–amyloid‐restricted AD population in scenario 5 escalated the ICER to CNY 1,122,389/QALY. General AD populations decline more slowly than strictly amyloid‐positive cohorts;^52^ consequently, this slower baseline progression inherently restricts the absolute QALY gains derived from lecanemab's relative treatment effect. This sensitivity highlights a key limitation: the lack of a biomarker‐confirmed Chinese natural history cohort necessitates our reliance on Western SoC data. Establishing domestic Aβ‐confirmed registries is essential to reduce structural uncertainties in future economic evaluations.

We found that, even when caregiver burden and long‐term care costs were incorporated from a societal perspective, the improvement in the cost‐effectiveness of lecanemab remained limited. Although long‐term care for patients with AD imposes substantial caregiving burden and productivity losses on families, the disease‐modifying benefits and delay in progression provided by lecanemab are still insufficient to fully offset its high drug costs. This finding is consistent with a study from South Korea, which reported that, after including caregiver burden under a limited societal perspective, the ICER decreased by only ≈ 8.6%, indicating a modest overall improvement.^19^

Notably, in China, most patients with AD are still primarily cared for at home, with a relatively low proportion entering institutional or long‐term care facilities.^53^ Due to traditional family‐based caregiving culture, limited availability of long‐term care resources, and the relatively high cost of institutional care, a large proportion of patients rely on informal care provided by family members. As a result, the disease burden is predominantly reflected in caregiver strain and indirect societal costs, including time loss from caregiving, labor force withdrawal, psychological burden, and reduced quality of family life.^9^ When monetized using the opportunity cost approach, this massive informal care burden causes the calculated societal cost of community care to exceed the out‐of‐pocket fees of institutional care.

However, the primary economic benefit of lecanemab does not come merely from keeping patients out of institutions, but from keeping them in milder disease stages for a longer duration. Informal care costs are strictly stage dependent; by delaying progression to severe AD when family caregiving costs reach their absolute peak, lecanemab offsets substantial societal opportunity costs. Consistent with this, a dynamic microsimulation study in the United States reported that lecanemab treatment over 4 years could generate ≈ USD 21,398 in combined societal value for patients and caregivers.^48^ Nevertheless, because many patients continue to be cared for primarily by family members even as the disease progresses, the therapy's ability to fully alleviate the long‐term caregiving burden remains limited under the current Chinese care model.^8^, ^44^ With the emergence of future DMTs offering improved long‐term efficacy and more convenient administration, the health economic value of anti‐amyloid therapies may further evolve.

The findings also suggest that China may need to further improve insurance coverage systems for innovative therapies for AD. A study in the United States showed that, under Medicare and supplemental commercial insurance coverage, most patients experienced substantially reduced out‐of‐pocket expenditures, whereas those without supplementary insurance were required to pay ≈ 20% of drug costs.^43^ In contrast, current reimbursement and commercial insurance coverage for lecanemab in China remains limited, which may further restrict patient access and increase the financial burden on families. Therefore, as DMTs gradually enter the Chinese market, establishing a sustainable payment system and risk‐sharing mechanisms will become an important public health issue.

This study has several strengths. First, it is the first to conduct a long‐term cost‐effectiveness analysis of anti‐amyloid monoclonal antibodies for AD based on real‐world data from China, providing a closer reflection of local clinical practice. Second, the model simultaneously incorporates dual dimensions of cognitive stage and care setting transitions, while also accounting for long‐term maintenance therapy, residual effects after treatment discontinuation, and caregiver disutility, thereby improving clinical realism. In addition, extensive sensitivity and scenario analyses were performed to systematically assess the robustness of the results.

This study also has several limitations. First, disease progression estimates are constrained by our cohort's limited sample size and short follow‐up. Crucially, lacking a biomarker‐confirmed Chinese natural history cohort, we relied on Western data for the SoC arm; this structural mismatch introduces uncertainty, as results are highly sensitive to natural history assumptions. Second, real‐world evidence regarding the long‐term efficacy of lecanemab, optimal treatment duration, and post‐discontinuation residual effects is still limited; therefore, some long‐term extrapolations rely on assumptions. Finally, health utilities and certain care costs were derived from international sources, and while justified for advanced AD, these proxy utility assessments may not capture quality of life as accurately as self‐reported valuations.

In conclusion, although lecanemab provides some clinical benefits for patients with early‐stage AD in China, its long‐term cost‐effectiveness remains unlikely to meet commonly used WTP thresholds under current pricing. Future improvements in economic value may depend on drug price reductions, optimization of reimbursement policies, and more precise patient selection strategies. With the accumulation of long‐term real‐world evidence, ongoing updates to the health economic evaluation of DMTs for AD will be warranted.

## 5 CONCLUSION

Lecanemab can provide certain health benefits in Chinese patients with early AD, but it does not yet demonstrate favorable cost‐effectiveness under the current pricing system. In the future, it is still necessary to further validate its long‐term economic value in combination with drug price reductions, improvements in the payment system, and the continuous follow‐up of domestic natural history and clinical cohorts.

## CONFLICT OF INTEREST STATEMENT

The authors declare no conflicts of interest. Author disclosures are available in the Supporting Information.

## CONSENT STATEMENT

The study was approved by the clinical research and application ethics committee of the Second Affiliated Hospital of Guangzhou Medical University (No. KY‐EC2024‐049‐01). All participants and study partners provided written consent.

## Supporting information



Supporting Information

Supporting Information
