# Supplementary material for: Cost‐effectiveness of lecanemab for early Alzheimer's disease in China
Source: Alzheimers Dement. 2026 Oct 27;22(10):e71941. doi: 10.1002/alz.71941 (PMC13714087; doi:10.1002/alz.71941)
Supplement: Supplementary file 2 [file ALZ-22-e71941-s002.docx]

**Supplementary Material**

**Cost-Effectiveness of Lecanemab for Early Alzheimer's Disease in China**

**[Supplemental S1. Modeling and Derivation of Dynamic Disease Progression and Multi-Phase Transition Probabilities for the Lecanemab Cohort	2](#_Toc24554)**

**[Supplemental Table 1. Base Annual Mortality Probabilities in China by Age Group	4](#_Toc30040)**

**[Supplemental Table 2. One-Way Sensitivity Analysis: Input Parameters and Their Impact on the Incremental Cost-Effectiveness Ratio (ICER)	5](#_Toc19693)**

**[Supplemental Table 3. Summary of Probabilistic Sensitivity Analysis Results	7](#_Toc29698)**

**[Supplemental S2. Scenario Analyses	8](#_Toc21751)**

**[Supplemental Table 4. Results of Scenario Analyses	12](#_Toc20588)**

**Supplemental S1. Modeling and Derivation of Dynamic Disease Progression and Multi-Phase Transition Probabilities for the Lecanemab Cohort**

In the Markov model, a two-phase dynamic treatment-effect framework was used to characterize the long-term effects of lecanemab. The framework incorporated Chinese real-world evidence (RWE) to estimate disease progression during active treatment, an amyloid-positive (Aβ+) natural history cohort[1] as the reference for standard-of-care (SoC) progression, and a treatment-waning effect after discontinuation.

Phase 1: Active Treatment Period

During active treatment, disease progression was estimated using a Chinese real-world cohort of 108 patients with MCI or mild AD who had at least two complete CDR-SB assessments. A linear mixed-effects model (LMM) with random intercepts and random slopes was used to characterize individual CDR-SB trajectories, accounting for between-patient differences in baseline disease severity and progression rates[2]. The LMM estimated a mean baseline intercept of 4.5416 CDR-SB points and an annual progression slope of 0.6264 points/year in the lecanemab-treated cohort.

The estimated CDR-SB trajectory was subsequently translated into discrete disease-stage transition probabilities for the 3-month Markov cycle. The time required to reach the prespecified CDR-SB threshold for progression from mild to moderate AD was derived from the estimated baseline score and annual progression slope, and the resulting time-to-progression was converted into an instantaneous transition rate and subsequently into a 3-month transition probability.

Mild-to-moderate AD progression was estimated at 3.11% per 3-month cycle during active treatment. Comparison with the corresponding Aβ+ SoC progression rate yielded an HR of 0.2510, corresponding to a 74.9% reduction in the relative risk of disease progression. This relative treatment effect was then applied to the MCI-to-mild AD SoC progression rate to derive the treatment-adjusted transition probability.

This approach incorporated treatment effects estimated from Chinese RWE while retaining the Aβ+ SoC population as the reference disease-progression framework.

Phase 2: Discontinuation Waning Period

Based on long-term follow-up reports from the phase 2 Study 201 open-label extension[3,4], the disease-delaying effect of lecanemab was assumed to persist after treatment discontinuation and gradually decline over time. Under the base-case assumption, the treatment effect declined linearly, reaching 39% of the active-treatment effect after 1 year and zero by the end of the second year[5].

To accurately simulate this complex transition, we applied a two-year linear waning integral weighting method to calculate the average residual efficacy during the waning period. Integral calculations yielded an overall average efficacy multiplier of 44.5% over the two years off-treatment. The adjusted disease delay rate during the waning period was calculated as 33.33%, corresponding to a weighted waning HR of 0.6667. After the two-year discontinuation buffer period expires, the residual protective effect is completely washed out, and the patient's transition probabilities fully revert to those of the SoC group.

**Supplemental Table 1. Base Annual Mortality Probabilities in China by Age Group**

| **Age Band (Years)** | **Base Annual Mortality Probability** |
| --- | --- |
| 50 – 54 | 0.00299 |
| 55 – 59 | 0.00451 |
| 60 – 64 | 0.00748 |
| 65 – 69 | 0.01172 |
| 70 – 74 | 0.02026 |
| 75 – 79 | 0.03563 |
| 80 – 84 | 0.06286 |
| 85 – 89 | 0.10276 |
| 90 – 94 | 0.16165 |
| 95 – 99 | 0.20695 |

**Supplemental Table 2. One-Way Sensitivity Analysis: Input Parameters and Their Impact on the Incremental Cost-Effectiveness Ratio (ICER)**

| **Parameter** | **Base Case** | **Lower Bound** | **ICER** | **Upper Bound** | **ICER** |
| --- | --- | --- | --- | --- | --- |
| Mean Weight (kg) | 59.49 | 42.76 | 254,925 | 76.22 | 483,352 |
| Starting Age (years) | 71 | 61.9 | 306,249 | 80.1 | 515,094 |
| Lecanemab Price / Vial | 2508 | 2006 | 287,848 | 3010 | 450,429 |
| SoC: Mild to Mod | 0.1182 | 0.0946 | 420,404 | 0.1418 | 338,529 |
| SoC: MCI to Mild | 0.0628 | 0.0502 | 418,039 | 0.0754 | 337,804 |
| Utility Comm: Mild | 0.922 | 0.738 | 419,711 | 0.999 | 351,419 |
| Utility Comm: MCI | 0.988 | 0.79 | 431,761 | 0.999 | 366,188 |
| SoC: Mod to Severe | 0.1256 | 0.1005 | 394,264 | 0.1507 | 352,296 |
| Utility Inst: Severe | 0.436 | 0.349 | 351,884 | 0.523 | 388,173 |
| Discount Rate | 0.05 | 0.04 | 353,077 | 0.06 | 385,596 |
| Utility Comm: Severe | 0.595 | 0.476 | 353,658 | 0.714 | 386,036 |
| Leca Active: MCI to Mild | 0.0162 | 0.0116 | 352,532 | 0.0206 | 384,876 |
| Discontinuation Rate | 0.0341 | 0.0273 | 384,309 | 0.0409 | 355,151 |
| Leca Active: Mild to Mod | 0.0311 | 0.0224 | 356,701 | 0.0395 | 381,012 |
| HR Death: Mild | 2.23 | 1.77 | 358,216 | 2.82 | 382,452 |
| Utility Comm: Mod | 0.821 | 0.657 | 358,693 | 0.985 | 380,211 |
| Utility Inst: Mild | 0.763 | 0.61 | 378,398 | 0.916 | 360,322 |
| Cost SoC Inst: Severe | 76569 | 61255 | 377,770 | 91883 | 360,507 |
| HR Death: Severe | 4.98 | 3.85 | 377,653 | 6.44 | 361,900 |
| Cost SoC Comm: Severe | 102985 | 82388 | 376,715 | 123582 | 361,563 |
| Utility Inst: MCI | 0.829 | 0.663 | 375,917 | 0.995 | 362,600 |
| Cost SoC Comm: Mild | 50474 | 40379 | 362,528 | 60569 | 375,749 |
| Caregiver Disutil: Severe | 0.086 | 0.069 | 375,078 | 0.103 | 363,385 |
| HR Death: MCI | 1.61 | 1.49 | 363,925 | 1.74 | 374,716 |
| Waning: Mild to Mod | 0.0805 | 0.0768 | 365,564 | 0.084 | 372,390 |
| Waning: MCI to Mild | 0.0423 | 0.0404 | 365,941 | 0.0442 | 372,247 |
| Waning: Mod to Severe | 0.0856 | 0.0817 | 365,922 | 0.0894 | 372,152 |
| Cost SoC Comm: MCI | 17161 | 13729 | 366,625 | 20593 | 371,653 |
| Cost SoC Comm: Mod | 64722 | 51778 | 371,437 | 77666 | 366,840 |
| Caregiver Disutil: Mild | 0.036 | 0.029 | 367,045 | 0.043 | 371,256 |
| Inst Prob: Severe | 0.0721 | 0.0577 | 370,650 | 0.0865 | 367,932 |
| Cost SoC Inst: Mild | 37527 | 30022 | 367,938 | 45032 | 370,339 |
| Inst Prob: Mild | 0.0095 | 0.0076 | 367,981 | 0.0114 | 370,245 |
| Inst Prob: MCI | 0.005 | 0.004 | 368,126 | 0.006 | 370,122 |
| Caregiver Disutil: Mod | 0.07 | 0.056 | 369,991 | 0.084 | 368,290 |
| Utility Inst: Mod | 0.662 | 0.53 | 369,775 | 0.794 | 368,505 |
| HR Death: Mod | 3.1 | 2.47 | 368,379 | 3.89 | 369,618 |
| MRI Monitoring Cost | 600 | 480 | 368,758 | 720 | 369,519 |
| Inst Prob: Mod | 0.0289 | 0.0231 | 369,515 | 0.0347 | 368,772 |
| Cost SoC Inst: MCI | 12759 | 10207 | 368,862 | 15311 | 369,416 |
| Cost SoC Inst: Mod | 48120 | 38496 | 369,013 | 57744 | 369,264 |
| Overall ARIA Prob | 0.092 | 0.0736 | 369,114 | 0.1104 | 369,164 |
| Severe ARIA Prob | 0.004 | 0.0032 | 369,130 | 0.0048 | 369,147 |
| Disutility: Severe ARIA | 0.14 | 0.112 | 369,130 | 0.168 | 369,147 |
| Baseline Inst Rate | 0.02 | 0.016 | 369,139 | 0.024 | 369,139 |

**Supplemental Table 3. Summary of Probabilistic Sensitivity Analysis Results**

| **Mean Cost** | **Lower CI** | **Upper CI** | **Mean QALY** | **Lower CI** | **Upper CI** | **Median ICER** |
| --- | --- | --- | --- | --- | --- | --- |
| 450,135 | 285,222 | 657,332 | 1.2289 | 0.7137 | 1.7508 | 366,433 |

**Supplemental S2. Scenario Analyses**

Given the remaining uncertainty surrounding the natural history of Alzheimer's disease (AD), the long-term effectiveness of disease-modifying therapies, and regional variations in health preferences and reimbursement policies, a series of scenario analyses were conducted in addition to deterministic and probabilistic sensitivity analyses. These analyses were designed to evaluate the robustness of the cost-effectiveness results under alternative structural assumptions and policy settings.

**Scenario 1: Alternative Treatment Durations**

The optimal duration of lecanemab therapy and the persistence of treatment effects after discontinuation remain uncertain. To explore the impact of treatment duration on long-term outcomes, fixed-duration treatment scenarios were evaluated. Lecanemab treatment was assumed to continue for 18 months (corresponding to the duration of the CLARITY AD trial), 3 years, and 4 years, respectively. After reaching the predefined treatment duration, patients discontinued therapy and transitioned to a post-treatment state in which treatment effects gradually waned over time. As a comparator, a lifetime-treatment scenario was also evaluated, assuming that patients remained on lecanemab throughout the entire simulation horizon regardless of disease progression.

**Scenarios 2 and 3: Alternative Baseline Disease States**

To assess the potential impact of earlier disease detection and treatment initiation, two alternative baseline cohort distributions were examined. In Scenario 2, all patients entered the model at the mild cognitive impairment (MCI) due to AD stage, whereas in Scenario 3, all patients entered at the mild AD dementia stage. These analyses aimed to explore the trade-off between greater long-term health gains achieved through earlier intervention and the additional treatment costs associated with prolonged survival and extended treatment exposure.

**Scenarios 4–6: Alternative Treatment Efficacy and Disease Progression Assumptions**

The base-case analysis incorporated stage-specific treatment effects derived from real-world evidence. To examine the influence of alternative efficacy and disease progression assumptions, three additional scenarios were evaluated.

Scenario 4 (Conservative Treatment Effect Assumption): A constant hazard ratio (HR = 0.69) derived from the CLARITY AD trial[6] was applied throughout the treatment period, representing the overall treatment effect reported in the pivotal clinical trial.

Scenario 5 (Alternative Standard-of-Care Progression Rates): Disease progression rates under standard of care (SoC) were replaced with transition probabilities reflecting the broader, non-biomarker-confirmed natural history of general AD, while the treatment effects of lecanemab remained unchanged. The MCI to mild AD transition rate was derived from a meta-analysis of 41 cohorts[7]. Subsequent transitions (mild to moderate to severe AD) were sourced from a 2012 study by Spackman et al. using the National Alzheimer's Coordinating Center database[8], which included patients aged ≥50 years (N = 3,852).

Scenario 6 (Alternative Progression Rates for Both Treatment Arms): Transition probabilities for both the SoC and lecanemab arms were replaced using estimates from alternative published studies to test a different model structure. Specifically, the alternative SoC progression matrix adopted a design that allows for state regression as well as cross-stage transitions[9]. To evaluate the impact of jointly modifying the disease progression structure and the treatment effect, the lecanemab arm in this scenario utilized the overall clinical hazard ratio (HR = 0.69) directly derived from the CLARITY AD trial[6].

**Scenarios 7 and 8: Alternative Structural Assumptions on Long-Term and Maintenance Efficacy**

To rigorously test whether the overarching economic conclusion relies on potentially optimistic structural inputs regarding long-term drug benefits and maintenance dosing, two conservative scenarios were introduced.

Scenario 7 (No Residual Benefit): It was assumed that there is absolutely no residual disease-delaying benefit upon treatment discontinuation. Transition probabilities immediately reverted to natural disease progression rates once therapy was stopped.

Scenario 8 (Halved Treatment Efficacy During Maintenance): It was assumed that halving the dosing frequency during the maintenance phase proportionally halved the drug's relative risk reduction (RRR) compared to the intensive dosing phase.

**Scenario 9: Survival Analysis-Derived Progression Probabilities**

To provide formal internal validation of the model against the cohort's actual observed progression, a scenario was run substituting the base-case Linear Mixed Model (LMM) parameters with raw, empirically observed transition rates, a 3-month transition probability of 3.15% for MCI to mild AD and 6.16% for mild to moderate AD These parameters were calculated via an empirical event-driven person-year approach defining progression as a strictly sustained event.

**Scenarios 10 and 11: Alternative Health-State Utility Sets**

Health-state utility values may vary across countries because of differences in population preferences, cultural perceptions of health, and long-term care systems. To evaluate the impact of alternative utility estimates on quality-adjusted life-year (QALY) outcomes, two additional utility sets were examined. Scenario 10 adopted EQ-5D utility values reported in a Korean study[10], while Scenario 11 employed utility estimates derived from a Swedish systematic review and meta-analysis[11]. These analyses assessed the sensitivity of model outcomes to different health preference weights.

**Scenario 12: Increased Institutional Care Utilization**

The initial proportion of patients receiving institutional care was increased from 2.00% to 9.13%, based on another proportion of Alzheimer’s disease patients receiving nursing-facility care reported in China[12]. Stage-specific 3month institutionalization probabilities were also updated to 0.00327, 0.01404, 0.03477, and 0.084 for MCI due to AD, mild AD dementia, moderate AD dementia, and severe AD dementia, respectively[13].

**Scenario 13: Insurance Reimbursement Policy**

To evaluate the effect of supplementary insurance coverage on patient affordability and cost-effectiveness outcomes, a reimbursement scenario based on the Guangzhou “Sui Sui Kang” commercial health insurance program was modeled[14]. Under this scenario, an annual deductible of CNY 16,000 was applied, followed by reimbursement of 50% of outpatient drug costs and 70% of inpatient drug costs. Based on observed treatment pathways, a weighted reimbursement rate was calculated and incorporated into the model. This analysis assessed the extent to which supplementary insurance coverage could reduce patient out-of-pocket expenditures and improve the cost-effectiveness of lecanemab treatment.

**Supplemental Table 4. Results of Scenario Analyses**

| **Strategy** | **Cost (CNY)** | **Incremental Cost (CNY)** | **Effectiveness (QALYs)** | **Incremental Effectiveness (QALYs)** | **ICER (CNY/QALY)** |
| --- | --- | --- | --- | --- | --- |
| **1. Treatment Duration** | | | | | |
| SoC | 360,776 | 4.3666 |  |  |  |
| Lecanemab: 18 months | 553,111.8 | 5.2091 | 192,335.8 | 0.8425 | 228,287.5 |
| Lecanemab: 3 years | 657,482.8 | 5.3717 | 296,706.8 | 1.0051 | 295,199.6 |
| Lecanemab: 4 years | 703,409.8 | 5.4439 | 342,633.8 | 1.0772 | 318,070.7 |
| Lecanemab: Lifetime | 913,393.9 | 5.6851 | 552,617.9 | 1.3185 | 419,129.5 |
| **2. Cohort Severity: 100% MCI** | | | | | |
| SoC | 308,289.3 | 5.2241 |  |  |  |
| Lecanemab | 858,362.8 | 6.7086 | 550,073.5 | 1.4845 | 370,554.6 |
| **3. Cohort Severity: 100% Mild AD** | | | | | |
| SoC | 409,419.7 | 3.5719 |  |  |  |
| Lecanemab | 964,395.7 | 4.7366 | 554,976 | 1.1647 | 476,508.8 |
| **4. Conservative Treatment Effect Assumption (Lecanemab HR = 0.69)** | | | | | |
| SoC | 360,776 | 4.3666 |  |  |  |
| Lecanemab (HR=0.69) | 726,228.7 | 4.8584 | 365,452.8 | 0.4918 | 743,141.8 |
| **5. Alternative SoC Progression** | | | | | |
| SoC | 308,921.4 | 5.6632 |  |  |  |
| Lecanemab | 812,251.9 | 6.1116 | 503,330.5 | 0.4484 | 1,122,389 |
| **6. Alternative Progression Rates (SoC and Lecanemab)** | | | | | |
| SoC | 358,652.4 | 4.3739 |  |  |  |
| Lecanemab | 727,943.5 | 4.7948 | 369,291.1 | 0.4208 | 877,521.5 |
| **7. No residual benefit** | | | | | |
| SoC | 360,776 | 4.3666 |  |  |  |
| Lecanemab | 833,019.5 | 5.3489 | 472,243.6 | 0.9823 | 480,752.9 |
| **8. Halved Treatment Efficacy During Maintenance** | | | | | |
| SoC | 360,776 | 4.3666 |  |  |  |
| Lecanemab | 790,086.3 | 5.3417 | 429,310.3 | 0.9751 | 440,273.1 |
| **9. Survival Analysis-Derived Progression Probabilities** | | | | | |
| SoC | 360,776 | 4.3666 |  |  |  |
| Lecanemab | 750,423.7 | 5.0455 | 389,647.8 | 0.6789 | 573,939.9 |
| **10. Health-State Utilities (South Korea utility set)** | | | | | |
| SoC | 360,776 | 2.0087 |  |  |  |
| Lecanemab | 824,004.30 | 2.9747 | 463,228.30 | 0.966 | 479,543.2 |
| **11. Health-State Utilities(Swedish utility set)** | | | | | |
| SoC | 360,776 | 3.4691 |  |  |  |
| Lecanemab | 824,004.30 | 4.5773 | 463,228.30 | 1.1082 | 418,019.1 |
| **12. Increased Institutional Care Utilization** | | | | | |
| SoC | 352,759 | 4.2930 |  |  |  |
| Lecanemab | 816,682 | 5.5276 | 463,923 | 1.2346 | 375,771.1 |
| **13. Insurance Reimbursement Policy** | | | | | |
| SoC | 360,776 | 4.3666 |  |  |  |
| Lecanemab | 594,089.10 | 5.6098 | 233,313.10 | 1.2432 | 187,676.9 |

**REFERENCE**

1. Potashman M, Buessing M, Levitchi Benea M, Cummings J, Borson S, Pemberton-Ross P, et al. Estimating Progression Rates Across the Spectrum of Alzheimer’s Disease for Amyloid-Positive Individuals Using National Alzheimer’s Coordinating Center Data. Neurol Ther 2021;10:941–53. https://doi.org/10.1007/s40120-021-00272-1.

2. Kansal AR, Tafazzoli A, Ishak KJ, Krotneva S, Collaboration A. Alzheimer’s disease Archimedes condition-event simulator: Development and validation. Alzheimer’s & Dementia: Translational Research & Clinical Interventions 2018;4:76–88. https://doi.org/10.1016/j.trci.2018.01.001.

3. Reyderman L, Bhagunde P, Penner N, Charil A, Dhadda S, Hersch S, et al. Lecanemab Maintenance Dosing in Early Alzheimer’s Disease: Support for Continued Dosing from Clinical, Pharmacology, and Modeling Data (S23.007). Neurology 2025;104:3687. https://doi.org/10.1212/WNL.0000000000211170.

4. McDade E, Cummings JL, Dhadda S, Swanson CJ, Reyderman L, Kanekiyo M, et al. Lecanemab in patients with early Alzheimer’s disease: detailed results on biomarker, cognitive, and clinical effects from the randomized and open-label extension of the phase 2 proof-of-concept study. Alz Res Therapy 2022;14:191. https://doi.org/10.1186/s13195-022-01124-2.

5. Whetten J, Dickson S, Duncan G, et al. Estimating the 10-year time-savings benefits of lecanemab treatment. Neurology. 2026;106(11_Supplement_1):4390. doi:10.1212/WNL.0000000000217107.

6. van Dyck CH, Swanson CJ, Aisen P, Bateman RJ, Chen C, Gee M, et al. Lecanemab in Early Alzheimer’s Disease. N Engl J Med 2023;388:9–21. https://doi.org/10.1056/NEJMoa2212948.

7. Mitchell AJ, Shiri-Feshki M. Rate of progression of mild cognitive impairment to dementia--meta-analysis of 41 robust inception cohort studies. Acta Psychiatr Scand 2009;119:252–65. https://doi.org/10.1111/j.1600-0447.2008.01326.x.

8. Spackman DE, Kadiyala S, Neumann PJ, Veenstra DL, Sullivan SD. Measuring Alzheimer disease progression with transition probabilities: estimates from NACC-UDS. Curr Alzheimer Res 2012;9:1050–8. https://doi.org/10.2174/156720512803569046.

9. Hoang MT, Kahn JG, Saha S, Trépel D. Value‐based prices of emerging disease‐modifying therapies for Alzheimer’s disease in 174 countries: a cost‐effectiveness and threshold analysis. Alzheimer’s &amp; Dementia 2026;22:e71308. https://doi.org/10.1002/alz.71308.

10. Shin S, Kim M, Hong SH. Economic Evaluation of Lecanemab for Early Symptomatic Alzheimer’s Disease in South Korea. Pharmacoecon Open 2025;9:793–804. https://doi.org/10.1007/s41669-025-00593-z.

11. Xia X, Aye S, Frisell O, Aho E, Handels R, Li Y, et al. The Cost-Effective Price of Lecanemab for Patients with Early Alzheimer’s Disease in Sweden. Pharmacoeconomics 2025;43:1251–66. https://doi.org/10.1007/s40273-025-01527-7.

12. Li Y, Leng F, Xiong Q, Zhou J, Du A, Zhu F, et al. Factors Associated With Alzheimer's Disease Patients' Caregiving Status and Family Caregiving Burden in China. Front Aging Neurosci. 2022 Mar 16;14:865933. doi: 10.3389/fnagi.2022.865933

13. Igarashi A, Azuma MK, Zhang Q, et al. Predicting the Societal Value of Lecanemab in Early Alzheimer’s Disease in Japan: A Patient-Level Simulation. Neurol Ther. 2023;12(4):1133–1157. doi:10.1007/s40120-023-00492-7

14. Guangzhou Municipal People's Government. 2026 Guangzhou “Suìsuìkāng” enrollment begins. Accessed August 20, 2026. [https://www.gz.gov.cn/zt/shb/content/post_10530156.html](https://www.gz.gov.cn/zt/shb/content/post_10530156.html).
